# Supplementary figures and images for: Comparison of Surgical Resection and Percutaneous Ultrasonographic Guided Radiofrequency Ablation for Initial Recurrence of Hepatocellular Carcinoma in Early Stage following Curative Treatment
Source: Cancers (Basel). 2022 Nov 10;14(22):5524. doi: 10.3390/cancers14225524 (PMC9688673; doi:10.3390/cancers14225524)

# Supplementary Figure S1

(a)

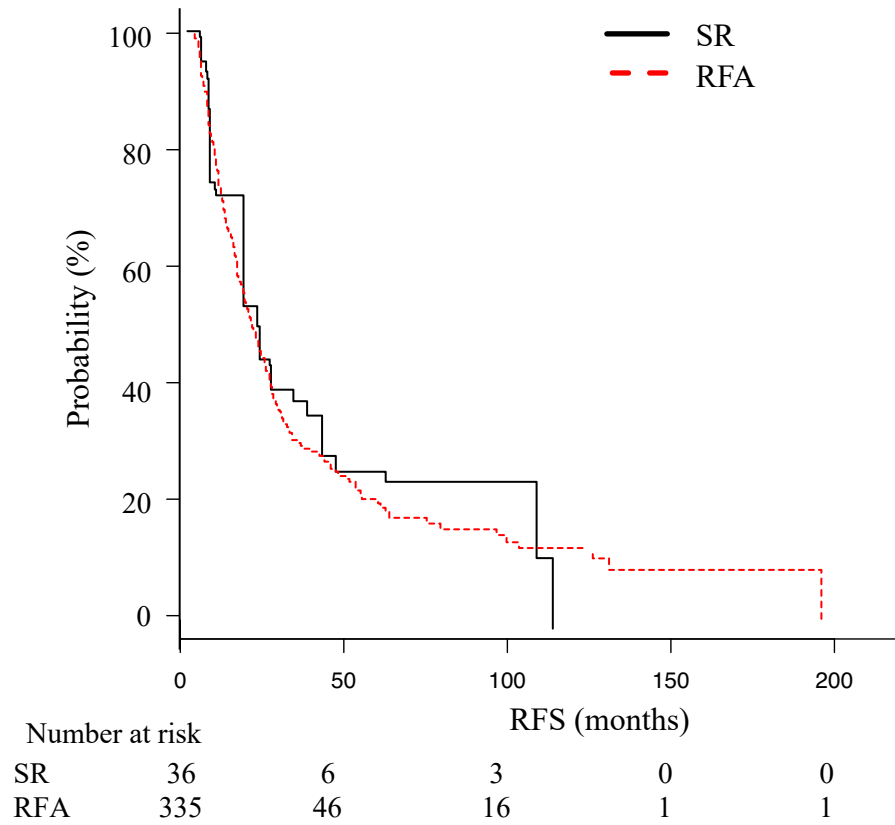

(b)

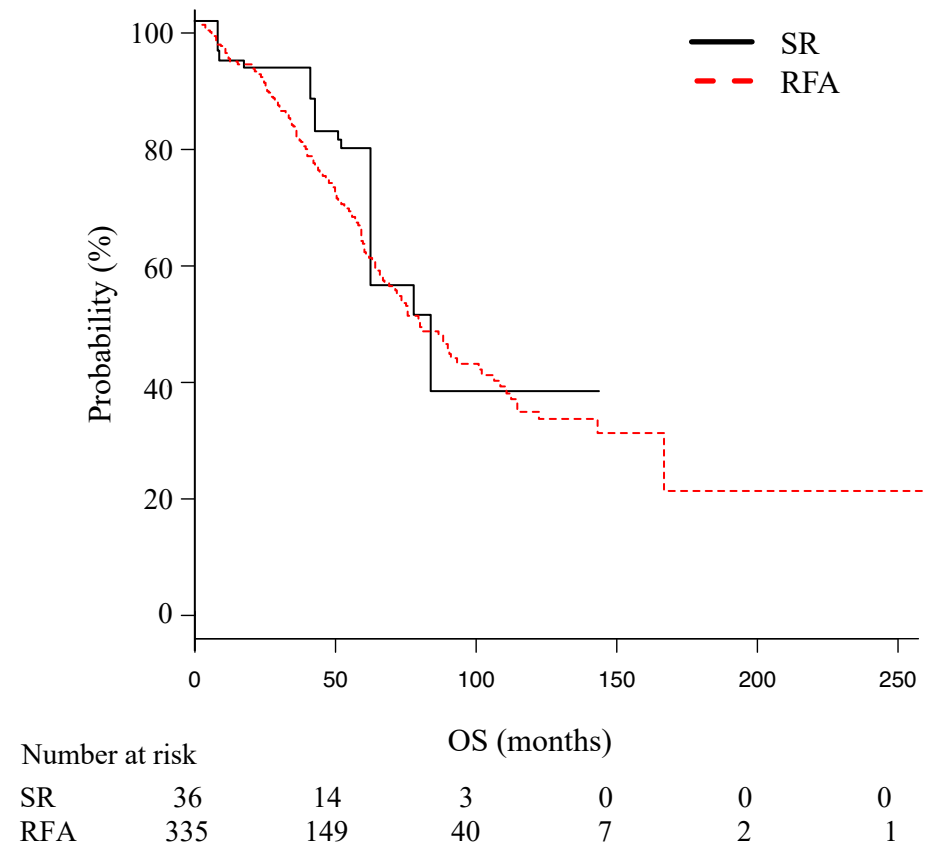

Supplement: Supplementary file 1 [file cancers-14-05524-s001.zip › cancers-2004285-supplementary.pdf]
